# A GP130-Targeting Small Molecule, LMT-28, Reduces LPS-Induced Bone Resorption around Implants in Diabetic Models by Inhibiting IL-6/GP130/JAK2/STAT3 Signaling

**DOI:** 10.1155/2023/9330439

**Published:** 2023-01-06

**Authors:** Qi-qi Liu, Wei-wei Wu, Jian Yang, Rui-bin Wang, Ling-ling Yuan, Pei-zhao Peng, Mao-yun Zeng, Ke Yu

**Affiliations:** The Affiliated Stomatological Hospital of Southwest Medical University, 2, Jiangyang Nan Road, Luzhou, China

## Abstract

In this study, we examined the effect of the GP130-targeting molecule, LMT-28, on lipopolysaccharide- (LPS-) induced bone resorption around implants in diabetic models using in vitro and rat animal experiments. First, LMT-28 was added to osteoblasts stimulated by LPS and advanced glycation end products (AGEs), and nuclear factor-*κ*B receptor-activating factor ligand (RANKL) and associated pathways were evaluated. Then, LMT-28 was administered by gavage at 0.23 mg/kg once every 5 days for 2 weeks to type 2 diabetic rats with peri-implantitis induced by LPS injection and silk ligature. The expression of IL-6 and RANKL was evaluated by immunohistochemistry, and the bone resorption around implants was evaluated by microcomputed tomography. The results showed that LMT-28 downregulated the expression of RANKL through the JAK2/STAT3 signaling pathway in osteoblasts stimulated by LPS and AGEs, reduced bone resorption around implants with peri-implantitis, decreased the expression of IL-6 and RANKL, and decreased osteoclast activity in type 2 diabetic rats. This study confirmed the ability of LMT-28 to reduce LPS-induced bone resorption around implants in diabetic rats.

## 1. Introduction

Peri-implantitis is an irreversible inflammatory change of hard and soft tissues around implants, which is caused by an imbalance of microorganisms in the peri-implant microenvironment [1]. The most typical feature of peri-implantitis is peri-implant bone loss (PBL), which causes the most serious damage to the implants. With the application of implants becoming more and more popular, it has been estimated that the incidence of peri-implantitis will grow rapidly in the coming decade [2]. Another chronic inflammatory disease, type 2 diabetes, increases each year with the aging of the population, which is characterized by a disorder of glucose metabolism [3]. According to current research, diabetes increases the susceptibility to peri-implantitis [4]. Therefore, there is an urgent need to prevent peri-implantitis, especially bone resorption, in patients with diabetes. First, we need to determine the mechanism of PBL in peri-implantitis in diabetes.

Based on existing research, the mechanism of bone loss in peri-implantitis has been explored from multiple angles. The host immune inflammatory response is considered to be the basic mechanism of peri-implant destruction in peri-implantitis [5]. This inflammatory response is stimulated by a bacterial biofilm, dental plaque, and plays a key role in PBL. Lipopolysaccharide (LPS), a toxin from bacteria (e.g., *Porphyromonas gingivalis*) in dental plaque, is shown to facilitate PBL due to a significant inflammatory response and persistent bone resorption activity [6]. In addition, in diabetes, rising blood glucose levels create advanced glycation end products (AGEs) that also lead to the higher expression of proinflammatory cytokines, such as interleukin-6 (IL-6) [7]. IL-6, an important inflammatory response factor, is secreted by multiple cells, including osteocytes and osteoblasts. When the secretion of IL-6 increases in some diseases associated with bone loss, such as periodontitis, peri-implantitis, and osteoporosis, binding of IL-6 to its receptor, IL-6R, promotes homodimerization of GP130, which then activates downstream signal transduction to cause bone resorption [8, 9]. IL-6 has been confirmed to induce osteoclast formation through an independent mechanism and has been shown to stimulate multiple cell types to express receptor activator of nuclear factor-*κ*B ligand (RANKL), which induces osteoclast precursors to become mature osteoclasts [10].

According to previous studies, LMT-28, a derivative of oxazolidinone, has been shown to significantly inhibit IL-6 activity through direct binding to GP130 and is nontoxic with oral availability [11]. LMT-28 alleviated collagen-induced arthritis and ameliorated the progression of pancreatitis in a mice model, and these two inflammatory diseases are related to an increase in IL-6 level [12]. This suggests that LMT-28 can be used to treat bone resorption in peri-implantitis, which is also a type of inflammation with increased IL-6 level. However, no research has yet confirmed this.

To evaluate the effects of LMT-28 on bone resorption in peri-implantitis, an appropriate animal model is needed that can simulate human diseases, diabetes mellitus, dentition defects, and peri-implantitis. In this study, we chose Sprague-Dawley (SD) rats, which are an ideal model for implant placement and induction of peri-implantitis [12–14]. Because osteoblasts express a large amount of IL-6 and RANKL in LPS-induced bone resorption, we chose osteoblasts as the cells to study in vitro [15]. The mechanism by which LMT-28 acts on GP130 to inhibit the effects of IL-6 on the downstream pathway of GP130 and the JAK2/STAT3 pathway was also verified in this study.

## 2. Materials and Methods

### 2.1. Porphyromonas Gingivalis Culture

*Porphyromonas gingivalis* (BNCC337441) obtained from BeNa Chuanglian Biotechnology Research Institute (Beijing, China) was grown at 37°C in brain heart infusion (BHI) broth medium in an anaerobic incubator. A bacterial LPS extraction kit (BestBio, Shanghai, China) was used to extract LPS from 50 to 200 mg of *P. gingivalis*, and the LPS concentration was measured by a total polysaccharide content measurement kit (Keming, Jiangsu, China).

### 2.2. LMT-28

LMT-28 (powder, HY-102084) was provided by MedChemExpress Inc. (NJ, USA). For in vitro experiments, LMT-28 was dissolved in dimethyl sulfoxide (DMSO, Solarbio, Beijing, China) to obtain a final concentration of 0.1–1,000 *μ*m. The volume concentration of DMSO in the cell culture medium was 0.1%. To examine its in vivo effects, LMT-28 was dissolved in 10% DMSO and 90% corn oil (MedChemExpress Inc., NJ, USA) at 2.5 mg/mL.

### 2.3. Cell Culture

Mouse embryonic osteoblasts (MC3T3-E1, Zhong Qiao Xin Zhou, Shanghai, China) were cultured in low-glucose Dulbecco's modified Eagle medium (DMEM, HyClone, Shanghai, China) supplemented with 10% fetal bovine serum (FBS, Tianhang, Hangzhou, China) containing 1% streptomycin/penicillin (Beyotime, Shanghai, China) and incubated in a humidified incubator (95% air and 5% CO_2_) at 37°C.

Various concentrations of LPS (1, 10, 100, 1,000, 10,000, and 100,000 ng/mL) were added to the osteoblasts, and the cells were harvested 2 h later. Real-time fluorescence quantitative PCR (RT-qPCR) was used to detect the expression of *RANKL* to determine the optimal stimulus concentration of LPS. Osteoblasts were treated with the optimal concentration of LPS and various concentrations (1, 10, 100, 1,000, 10,000, and 100,000 pg/mL) of AGEs (Cloud-Clone, TX, USA). After 2 h, the expression of RANKL was measured by RT-qPCR to determine the optimal concentration of AGEs and LPS. Then, osteoblasts were stimulated by the optimal concentration of LPS and AGEs for different periods (0, 0.5, 1, 2, 4, 6, 12, 24, and 48 h). Western blot (WB) and RT-qPCR were used to measure the expression of RANKL to determine the time at which RANKL expression was upregulated by LPS and AGEs and peaked. In addition, various concentrations of LMT-28 (0.1, 1, 10, 100, 1,000, and 10,000 nm) were added to the osteoblasts, and the cells were collected 2 h after stimulation. The expression of *RANKL* was measured by RT-qPCR to obtain the optimal stimulus concentration of LMT-28. The optimal concentration of LMT-28 was added to osteoblasts, and cells were harvested at different times (0, 0.5, 1, 2, 4, 6, 12, 24, and 48 h). The expression of RANKL was measured by RT-qPCR and WB for determining the optimal stimulation time of LMT-28.

The optimal stimulus concentration of dexamethasone (Macklin, Shanghai, China) and baricitinib (a JAK2 inhibitor, Beyotime, Shanghai, China) was evaluated by CCK8. Then, dexamethasone and LMT-28 were added to the cultured osteoblasts at their respective optimal concentrations. After the optimal stimulus time, the osteoblasts were harvested; the *RANKL* mRNA expression levels were evaluated using RT-qPCR; and RANKL protein, JAK2 phosphoprotein, and STAT3 phosphoprotein expression levels were evaluated using WB. In addition, the optimal concentrations of LPS, AGEs, baricitinib, and LMT-28 were added to the cultured osteoblasts. After the optimal stimulus time, the osteoblasts were harvested; the *RANKL* mRNA expression levels were evaluated using RT-qPCR; and RANKL protein, GP130 phosphoprotein, JAK2 phosphoprotein, and STAT3 phosphoprotein expression levels were determined using WB.

### 2.4. Animal Experiment

Eight-week-old male SD rats (*n* = 30, 241–282 g) were obtained from the Laboratory Animal Center of Southwest Medical University. The rats were fed in a ventilated room with a high-fat and high-sugar diet and water ad libitum. All surgical procedures were preapproved by the Animal Ethics Committee of Southwest Medical University (SWMU20210414).

**Figure 1:**
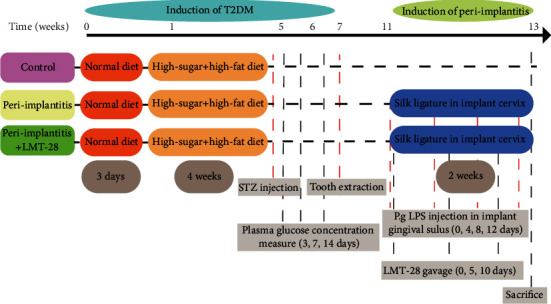
The vivo experiment protocol. Indicating specific procedures performed at each time interval.

After 4 weeks on a high-fat and high-sugar diet, all rats were injected intraperitoneally with streptozotocin (STZ) (30 mg/kg) to induce diabetes mellitus type 2 (T2DM). The body weight was recorded every week, and the plasma glucose concentration was measured at 3, 7, and 14 days postoperatively. Rats with three plasma glucose concentration measurements >16.7 mmol/L accompanied by weight loss were considered T2DM rats. The procedure and group assignment for the in vivo experiment are shown in Figure 1.

**Figure 2:**
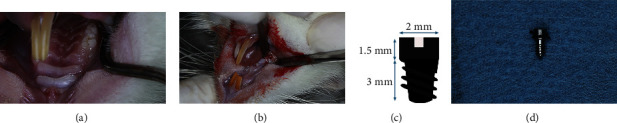
(a) Intraoral image after 4 weeks of natural healing after tooth extraction. (b) Intraoral image after the implant socket preparation during implant implantation. (c) The custom-made Ti–6AL–4 V screw-type implant. (d) 4-0 prelated silk wires before implant implantation.

Subsequently, the maxillary right first molar was extracted under general anesthesia with isoflurane (Figure 2(a)). After 4 weeks, first, the gingiva was cut from the top of the alveolar ridge at the maxillary right first molar region; then, mucoperiosteal flaps were elevated; and then, a hole was prepared with a 1.6 mm diameter reamer under saline irrigation (Figure 2(b)). Then, a custom-made Ti-6AL-4 V implant was inserted into the alveolar bone. The implant had a smooth top (2 mm diameter and 1.5 mm length) for gingival perforation and a screw-type root (1.8 mm diameter and 3 mm length) for osseointegration (Figure 2(c)). A random number table was used to allocate the animals to three groups as follows: control group, peri-implantitis group, and peri-implantitis+LMT-28 group.

Peri-implantitis was induced by an implant cervical ligature with 4-0 silk and implant gingival sulcus injection with *P. gingivalis* LPS. A 10 *μ*L *P. gingivalis* LPS (0.1 mg/mL) suspension was injected three times consecutively at 10 min intervals for each treatment (Figure 2(d)). This treatment was repeated every 4 days for a total of 2 weeks. Only saline was injected into the sulcus of implants in the control group. In peri-implantitis+LMT-28 group, LMT-28 (0.23 mg/kg) was given by gavage once every 5 days for a total of 2 weeks. Only saline was given by the same method to the other two groups.

### 2.5. CCK8 Assay

To measure cell proliferation, osteoblasts (1 × 10^4^ cells/well) were seeded into 96-well plates in 100 *μ*L of plain medium, treated with dexamethasone (1–100,000 nmol/L, serial dilution) or baricitinib (0.1–10,000 nmol/L, serial dilution), and incubated for 1, 3, 5, or 7 d. Subsequently, 10 *μ*L of CCK8 solution (Solarbio) was added to each well followed by incubation at 37°C in a 5% CO_2_ incubator for 1 h, and the absorbance was immediately measured at 450 nm.

### 2.6. RT-qPCR

Total RNA was extracted from mouse osteoblasts using a nonchloroform RNA extraction kit (Bioteke, Beijing, China) following the manufacturer's protocol. An aliquot of total RNA was reverse transcribed using a ReverTra Ace qPCR RT kit (TOYOBO, Tokyo, Japan) and amplified using SYBR Green real-time PCR master mix (TOYOBO). *GAPDH* (the internal control) expression levels were quantified and used to normalize gene expression levels. The 2^−∆∆CT^ method was used to analyze relative gene expression. Each experiment was replicated at least three times. RT-qPCR was performed with the following primers: *RANKL* (5′-CTGATGAAAGGAGGGAGCACG-3′ for sense and 5′-CTGATGAAAGGAGGGAGCACG-3′ for antisense) and *GAPDH* (5′-CCACTGGCGTCTTCACCAC-3′ for sense and 5′-CCTGCTTCACCACCTTCTTG-3′ for antisense).

### 2.7. WB Assay

Osteoblast cells were seeded into six-well plates at a density of 1 × 10^6^ cells/well. After stimulation was complete, cells were treated with RIPA lysis buffer (Bioteke) with 1 mm phenylmethylsulfonyl fluoride (PMSF) (Solarbio), 1% phosphatase inhibitors (Roche, Basel, Switzerland), and 1% proteinase inhibitor (Roche) for 30 min on ice. Cell lysates were resolved using 8–15% sodium dodecyl-sulfate polyacrylamide gel electrophoresis followed by transfer to polyvinylidene fluoride (PVDF) membranes. Membranes were then blocked with skim milk (Solarbio) at room temperature for 1 h and then incubated with the appropriate primary antibodies against RANKL, GAPDH, p-GP130, p-STAT3, STAT3, p-JAK2, or JAK2 (Affinity, USA) on an orbital shaker at 4°C overnight. Then, membranes were washed (5 min, three times) with tris-buffered saline with 0.1% Tween and incubated for 1 h with horseradish peroxidase-conjugated secondary antibodies diluted 1: 6,000. After washing (5 min, three times), membranes were incubated with an enhanced chemiluminescence reagent (Affinity), and the chemiluminescent signals were visualized using an iBright CL1000 imaging system (Thermo, MA, USA). The semiquantitative analysis was performed using ImageJ v1.8.0 (NIH, MD, USA).

### 2.8. Micro-Computed Tomography (CT) Analysis

Micro-CT (Inveon PET CT, Siemens, Germany) was performed to examine the bone absorption height (BAH) and bone absorption volume (BAV) using the following specifications: spot size, 50 *μ*m; tube voltage, 80 kVp; tube current, 500 *μ*A; and total rotation, 360°. After reconstructing the region of interest in three dimensions, the BAH was evaluated in sagittal sections of the implants as the distance from the implant platform to the first bone of implant contact. In addition, Inveon Research Workplace was used to calculate the BAV, which was defined as bone loss within 1 mm around the implant.

### 2.9. Histological and Immunohistochemical Analysis

The harvested maxillary alveolar bones were fixed in 4% paraformaldehyde and then decalcified in 10% ethylene diamine tetraacetic acid (EDTA) (Solarbio). The EDTA solution was replaced every 5 days for 5 weeks. Samples were embedded in paraffin after unscrewing the implants. The central part of the specimens was sectioned into 7 mm thick slices in the mesiodistal direction. Slides were stained using tartrate-resistant acid phosphatase (TRAP) staining according to the manufacturer's directions (BestBio). The slides were incubated with the primary antibodies overnight at 4°C after incubating in a goat serum-blocking solution for 20 min. Subsequently, the slides were washed with phosphate-buffered saline (PBS) three times for 5 min each time before staining with the secondary antibodies for 1 hr. The slides were washed with PBS three times for 5 min each time again. After incubating in DAB chromogenic solution for a few minutes, slides were counterstained with hematoxylin for 3 min. The investigator was blinded to the immunopositive outcome when photographing the slides using a camera system (Olympus BX51, Tokyo, Japan), and semiquantitative analysis was performed using ImageJ v1.8.0 (NIH).

### 2.10. Statistical Analysis

Statistical analyses were performed using SPSS statistical software (version 26.0; SPSS Inc., Chicago, IL, USA). Data were presented as means ± standard deviation (SD). Differences between groups were analyzed using one-way ANOVA, and least significant difference tests were used for post hoc tests. Statistical differences were considered significant at *p* < 0.05.

## 3. Results

### 3.1. The Optimal Concentration and Time for LPS, AGEs, and LMT-28 Osteoblast Treatment

**Figure 3:**
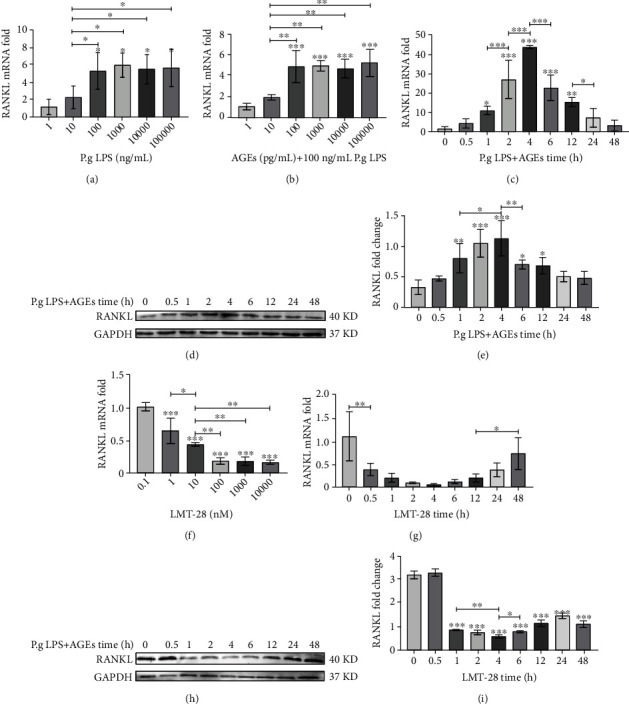
(a) The effect of different LPS concentrations on the expression of RANKL mRNA in osteoblasts. (b) The effect of 100 mg/mL LPS and AGEs on the expression of RANKL mRNA in osteoblasts. (c) The effects of LPS and AGE stimulation at optimum concentration for different times on RANKL mRNA expression in osteoblasts. (d, e) The effects of LPS and AGE stimulation at optimum concentration for different times on the expression of RANKL protein in osteoblasts (repeat three times). (f) The effect of LMT-28 on RANKL mRNA expression in osteoblasts. (g) The effects of stimulation at optimal LMT-28 concentration for different time on RANKL mRNA expression in osteoblasts. (h, i) The effects of stimulation at LMT-28 optimal concentration for different time on the expression of RANKL protein in osteoblasts (repeat three times; ^∗^*p* < 0.05, ^∗∗^*p* < 0.01, ^∗∗∗^*p* < 0.001).

With an increase in LPS concentration, the mRNA expression of *RANKL* in osteoblasts increased significantly (*p* < 0.05), but when the LPS concentration was higher than 100 ng/mL, *RANKL* expression tended to be constant, so the optimal concentration of LPS to stimulate *RANKL* expression in osteoblasts was set to 100 ng/mL (Figure 3(a)). The mRNA expression of *RANKL* in osteoblasts increased significantly with an increase in the AGE concentration (*p* < 0.05), but when the concentration of AGEs was higher than 100 pg/mL, the expression of *RANKL* tended to be constant. Therefore, the optimal concentration of AGEs to stimulate the expression of RANKL in osteoblasts was chosen as 100 pg/mL (Figure 3(b)). When osteoblasts were stimulated with 100 ng/mL LPS and 100 pg/mL AGEs, the mRNA and protein expression of RANKL increased and then peaked at 4 h, and then gradually decreased (Figures 3(c)–3(e)). The mRNA expression of *RANKL* in osteoblasts decreased significantly with an increase in the LMT-28 concentration (*p* < 0.05), but when the concentration of LMT-28 was higher than 100 nm, the expression of RANKL tended to be constant. Therefore, the optimal concentration of LMT-28 to attenuate the expression of RANKL in osteoblasts was 100 nm (Figure 3(f)). When osteoblasts were stimulated with 100 nm LMT-28, the mRNA and protein expression of RANKL decreased and reached a minimum at 4 h, and then gradually increased (Figures 3(g) and 3(h)).

### 3.2. The JAK2/STAT3 Pathway Is Involved in the Expression of RANKL in Osteoblasts

**Figure 4:**
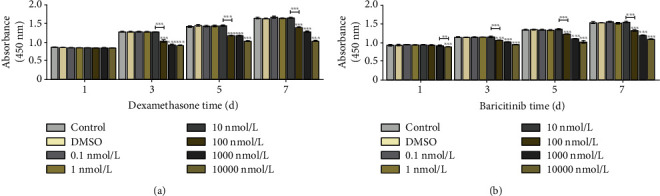
CCK8 assay results. (a) The effect of different concentrations of dexamethasone on osteoblast proliferation. (b) The effect of different concentrations of baricitinib on osteoblast proliferation (^∗∗^*p* < 0.01, ^∗∗∗^*p* < 0.001).

When dexamethasone was added to osteoblasts at different concentrations, there was no significant change in osteoblast proliferation at any concentration after 1 day (*p* > 0.05). However, 1,000 nmol/L, 10,000 nmol/L, and 100,000 nmol/L dexamethasone at 3, 5, and 7 days, respectively, significantly reduced the proliferation activity of osteoblasts (*p* < 0.05), while 100 nmol/L dexamethasone had no effect (*p* > 0.05, Figure 4(a)). When different concentrations of baricitinib were added to osteoblasts, it was found that 100 nmol/L, 1,000 nmol/L, and 10,000 nmol/L baricitinib at 3, 5, and 7 days, respectively, significantly reduced the proliferation activity of osteoblasts (*p* < 0.05), while 10 nmol/L baricitinib had no effect on the proliferation of osteoblasts (*p* < 0.05, Figure 4(b)).

**Figure 5:**
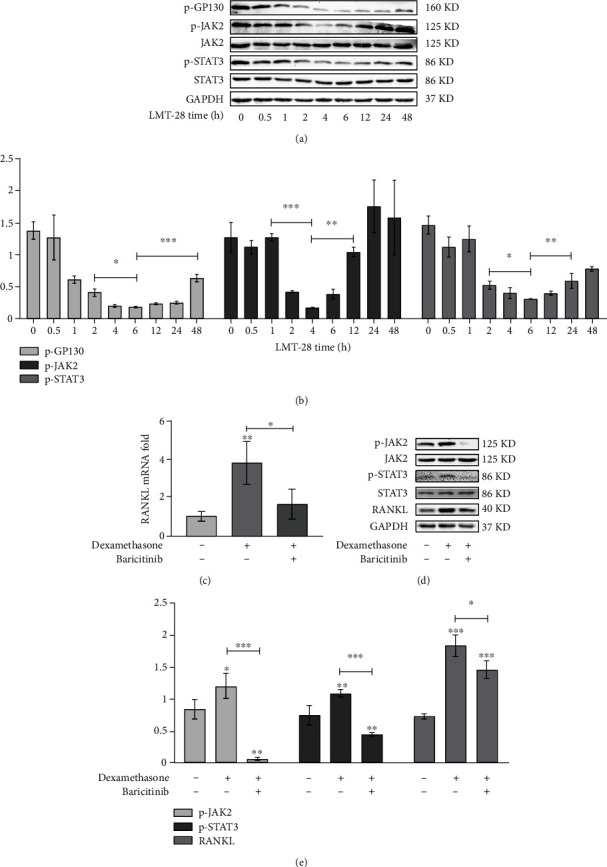
(a, b) The effect of LMT-28 at optimal concentration on JAK2-STAT3 and GP130 phosphorylation of osteoblasts at different times of stimulation (repeat three times). (c) The effect of dexamethasone and baricitinib on RANKL mRNA expression in osteoblasts. (d, e) The effect of dexamethasone and baricitinib on the expression of RANKL protein and the phosphorylation of JAK2-STAT3 pathway in osteoblasts (repeat three times; ^∗^*p* < 0.05, ^∗∗^*p* < 0.01, ^∗∗∗^*p* < 0.001).

Under LMT-28 stimulation, the phosphorylation levels of GP130, JAK2, and STAT3 in osteoblasts first decreased and then increased with time, and their expression trends were consistent with that of RANKL (Figures 5(a) and 5(b)). Dexamethasone treatment at 100 nmol/L upregulated the mRNA expression of *RANKL* in osteoblasts, but the mRNA expression of *RANKL* decreased significantly after adding 10 nmol/L baricitinib (*p* < 0.05, Figure 5(c)). Treatment of 100 nmol/L dexamethasone also upregulated the levels of RANKL protein, JAK2 phosphorylation, and STAT3 phosphorylation in osteoblasts, while the addition of 10 nmol/L baricitinib reduced the levels of RANKL protein, JAK2 phosphorylation, and STAT3 phosphorylation (Figures 5(d) and 5(e)).

### 3.3. Effects of LMT-28 on RANKL Expression and Related Signaling Pathways in Osteoblasts under LPS and AGE Costimulation

**Figure 6:**
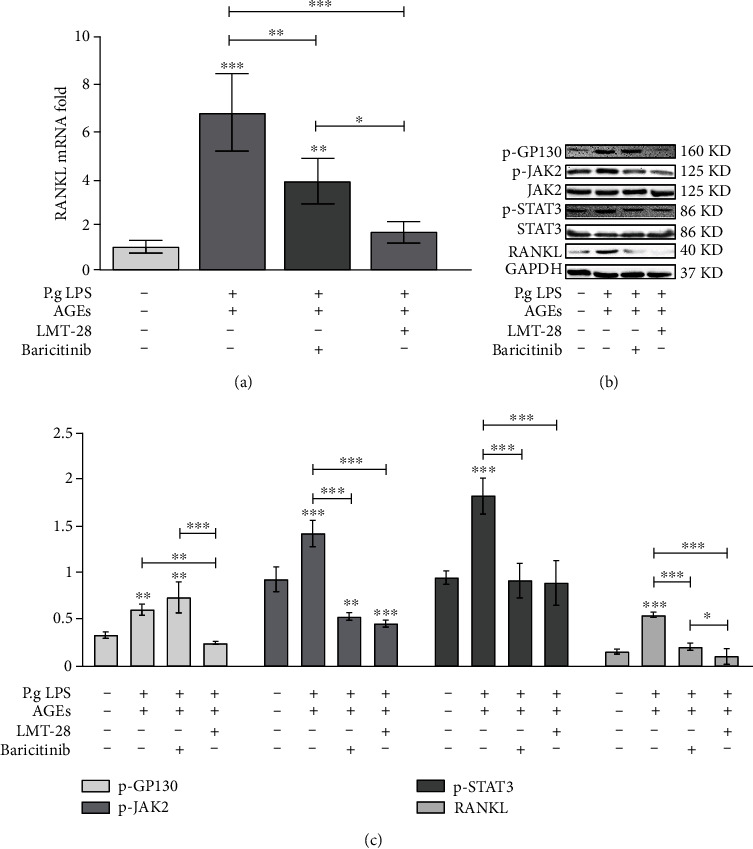
(a) The effect of LMT-28 on RANKL mRNA expression of osteoblasts under LPS and AGEs costimulation. (b, c) The effect of LMT-28 on the expression of RANKL protein and the phosphorylation levels of GP130, JAK2, and STAT3 in osteoblasts under LPS and AGEs costimulation and channel blocker baricitinib (repeat three times; ^∗^*p* < 0.05, ^∗∗^*p* < 0.01, ^∗∗∗^*p* < 0.001).

As shown in Figure 6(a), the simultaneous addition of 100 ng/mL LPS and 100 pg/mL AGEs significantly upregulated the expression of *RANKL* mRNA in osteoblasts, and the addition of 100 nmol/L LMT-28 or 10 nmol/L baricitinib significantly downregulated the mRNA expression of *RANKL* in osteoblasts under LPS and AGEs costimulation. However, 100 nmol/L LMT-28 restored the mRNA expression of *RANKL* in osteoblasts under LPS and AGE costimulation to normal levels, while the mRNA expression of *RANKL* in osteoblasts under LPS and AGE costimulation was still higher than that in the control group after adding 10 nmol/L baricitinib. Synergistic stimulation of 100 ng/mL LPS and 100 pg/mL AGEs upregulated the expression of RANKL protein and the phosphorylation levels of GP130, JAK2, and STAT3 in osteoblasts. After the addition of 10 nmol/L baricitinib, the protein expression of RANKL and the phosphorylation levels of JAK2 and STAT3 decreased, while the phosphorylation levels of GP130 remained unchanged. Furthermore, the addition of 100 nmol/L LMT-28 reduced the phosphorylation levels of JAK2 and STAT3, which was not significantly different than adding baricitinib. However, the protein expression of RANKL and phosphorylation levels of GP130 were significantly decreased following the LMT-28 treatment compared with baricitinib treatment (Figures 6(b) and 6(c)).

### 3.4. Plasma Glucose Levels and Body Weight

**Figure 7:**
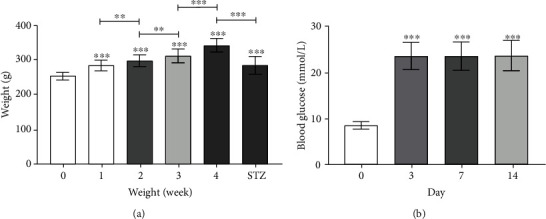
(a) The weight of SD rats after feeding for 0, 1, 2, 3, and 4 weeks and intraperitoneal injection of 1% STZ solution for 2 weeks. (b) The random blood glucose values of SD rats before intraperitoneal injection of 1% STZ solution and on days 3, 7, and 14 after injection (^∗∗^*p* < 0.01, ^∗∗∗^*p* < 0.001).

Rats fed a high-fat and high-sugar diet gained weight gradually and showed significantly lower weight gain after intraperitoneal injection of 1% STZ (Figure 7(a)). In addition, the presence of polyphagia, polydipsia, polyuria, and increased random blood glucose (>16.7 mmol/L) were observed in STZ-induced T2DM rats (Figures 7(b) and Table 1).

### 3.5. Characterizing Soft Tissue Degeneration in the Peri-Implant Region

**Figure 8:**
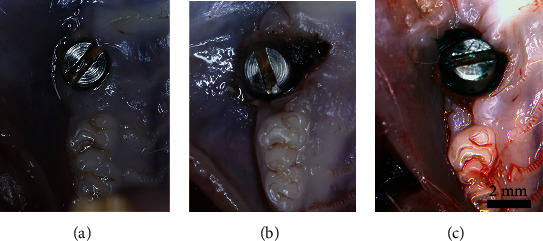
The intraoral observation results. (a) Control group. (b) Peri-implantitis group. (c) Peri-implantitis+LMT-28 group.

Two weeks after surgery, the soft tissue around the implants of the control group healed well and adhered closely to the gum penetrating part of the implant, with significantly less food residue and plaque around the implant, no obvious swelling, bleeding or pus in the gums, and no loosening or shedding of the implant (Figure 8(a)). In contrast, there was an obvious accumulation of food residue and plaque, poor soft tissue healing, gingival swelling, and obvious inflammation in the peri-implantitis group and peri-implantitis+LMT-28 group (Figures 8(b) and 8(c)). Five implants were loose in the peri-implantitis group, and one implant was loose in the peri-implantitis+LMT-28 group.

### 3.6. Characterizing Hard Tissue in the Peri-Implant Region

**Figure 9:**
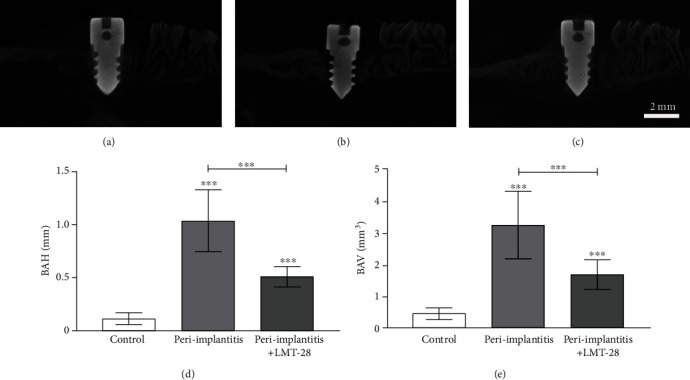
The micro-CT results. (a) Control group. (b) Peri-implantitis group. (c) Peri-implantitis+LMT-28 group. (d) The statistical analysis of implant BAH. (e) The statistical analysis of implant BAV (*n* = 10 rats per group; ^∗∗∗^*p* < 0.001).

Micro-CT was used to quantitatively evaluate and analyze bone resorption around the implants 2 weeks after surgery. All three groups had a certain amount of bone resorption around the implants, and the bone implant contact area was reduced, presenting a typical shallow disk or crater-like bone resorption (Figures 9(a)–9(c)). The BAH of the peri-implantitis group (1.04 ± 0.29 mm) and peri-implantitis+LMT-28 group (0.51 ± 0.10 mm) was significantly higher than that of the control group (0.12 ± 0.05 mm) (*p* < 0.05), and the BAH in the LMT-28 group was lower than that in the peri-implantitis+LMT-28 group (*p* < 0.05, Figure 9(d)). The BAV of the peri-implantitis group (3.23 ± 1.05 mm^3^) and peri-implantitis+LMT-28 group (1.69 ± 0.47 mm^3^) was significantly higher than that of the control group (0.46 ± 0.19 mm^3^) (*p* < 0.05), while the BAV of the peri-implantitis+LMT-28 group was significantly lower than that of the peri-implantitis group (*p* < 0.05, Figure 9(e)).

### 3.7. The Expression Level of IL-6 and RANKL and Osteoclast Activity in the Peri-Implant Region

**Figure 10:**
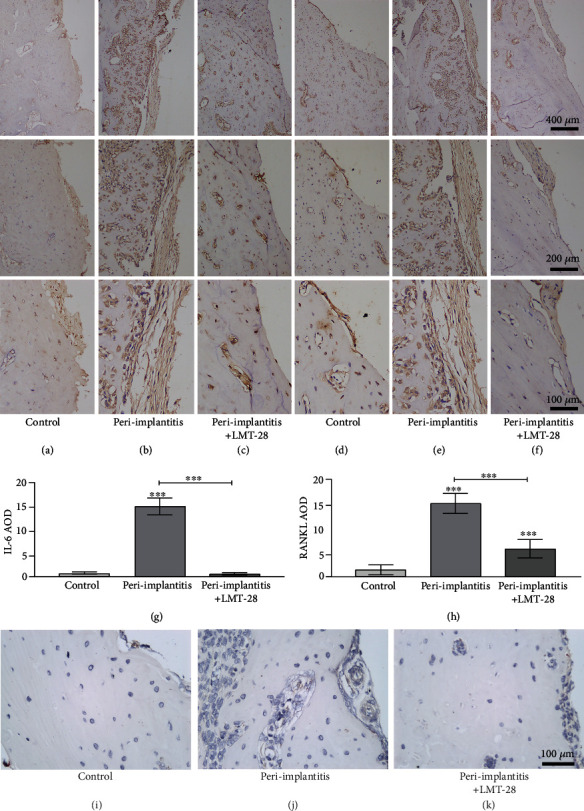
The histological and immunohistochemical results. (a–c) The expression of IL-6 in different treatment groups. (d–f) The expression of RANKL in different treatment groups. (g, h) The statistical analysis results of IL-6 and RANKL, respectively (*n* = 10 rats per group). (i–k) The TRAP staining results, (i) control group, (j) peri-implantitis group, (k) peri-implantitis+LMT-28 group (^∗∗∗^*p* < 0.001).

Immunohistochemical quantitative evaluation and analysis of IL-6 and RANKL expression in the peri-implant bone tissue showed that IL-6 expression in the peri-implantitis group was significantly higher than that in the control and the peri-implantitis+LMT-28 treatment groups (*p* < 0.05), while there was no significant difference in IL-6 expression between the peri-implantitis+LMT-28 group and the control group (*p* > 0.05, Figures 10(a)–10(c) and 10(g)). The expression level of RANKL in the peri-implantitis+LMT-28 and the peri-implantitis groups was significantly higher than that in the control group (*p* < 0.05), and the expression of RANKL in the peri-implantitis+LMT-28 group was significantly lower than that in the peri-implantitis group (*p* < 0.05, Figures 10(d)–10(f) and 10(h))). The TRAP staining results showed that osteoclast activity in the peri-implantitis group was increased, and that LMT-28 treatment inhibited osteoclast activity (Figures 10(i)–10(k)).

## 4. Discussion

**Figure 11:**
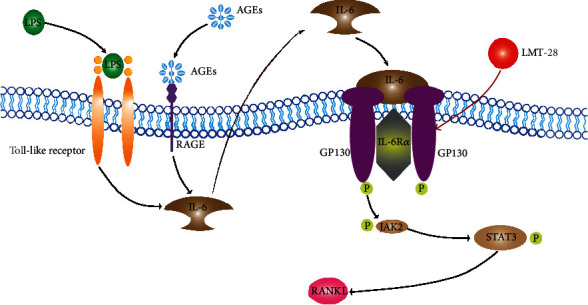
The signaling pathway representation of the results of the current study.

In this study, we demonstrated that LMT-28 ameliorated peri-implant bone absorption in a T2DM rat model with peri-implantitis during the healing period. In addition, LMT-28 suppressed *RANKL* expression in osteoblasts under LPS and AGE costimulation through the JAK2/STAT3 signaling pathway (Figure 11). Based on these results, we concluded that LMT-28 induced positive effects on tissue repair in a diabetic rat model with peri-implantitis during the healing period.

In the gingival crevicular fluid of patients with peri-implantitis, a variety of inflammatory cytokines are elevated, such as IL-1, IL-6, prostaglandins, and metalloproteinases [16]. Among them, the IL-6 family has been a research hotspot in recent years and has been identified as the classic bone resorption proinflammatory cytokine and an important mediator of pathological bone loss in peri-implantitis, although IL-6 is a double-edged sword in bone metabolism [17, 18]. According to the existing research, IL-6 promotes bone resorption in two ways. First, IL-6 induces the osteoblast line cell to express RANKL, which in turn stimulates osteoclast precursors into mature osteoclasts [19]. Second, IL-6 directly stimulates osteoclasts and participates in the differentiation and function of osteoclasts [20]. Therefore, some studies believe that inhibiting the high expression of IL-6 or decreasing the function of IL-6 will reduce bone resorption [21].

Multiple stimuli can increase the expression of IL-6, such as LPS and AGEs. LPS is a major pathogenic factor for peri-implantitis, which is produced by periodontal pathogens, including *P. gingivalis* [22]. LPS binds to TLR4, the membrane receptor of fibroblasts, osteoblasts, lymphocytes, and other cells; activates multiple signal pathways; and overexpresses multiple inflammatory factors, including IL-6 [23]. AGEs, substances in the blood of people with diabetes, also stimulate cells to express inflammatory cytokines such as IL-6 [24]. In this study, we again verified that costimulation by LPS and AGEs increased the expression of IL-6 in osteoblasts, and the secreted IL-6 further stimulated osteoblasts to express RANKL. Further, we verified that the JAK2/STAT3 signaling pathway was involved in the process of IL-6 stimulating osteoblasts to express RANKL (Figure 11). This evidence once again indicates the potential of inhibiting IL-6 to reduce bone resorption.

LMT-28 is one type of IL-6 inhibitor and acts by binding directly to GP130 and selectively inhibiting IL-6-induced phosphorylation of GP130, which is a common receptor and signal transducer for the IL-6 family of cytokines. To date, it has been reported that LMT-28 was used to treat many diseases with positive results [25–29]. Several studies reported that LMT-28 played a crucial role in the improvement of inflammation in collagen-induced arthritis by inhibiting the IL-6/GP130 signaling pathway [12, 26, 30]. In addition, LMT-28 was demonstrated to prevent impairment of cerebral autoregulation and hippocampal CA1 and CA3 neuronal necrosis after porcine fluid percussion brain injury [31]. LMT-28 inhibited the downstream signaling pathway of IL-6. In LPS-induced bone resorption, RANKL, a major contributor to the induction of mature osteoclasts [32], is the major downstream product of IL-6. In this study, we showed that LMT-28 reduced RANKL expression in osteoblasts stimulated by LPS and AGEs and in bone tissue with peri-implantitis in a T2DM rat model. LMT-28 also affected the IL-6 expression in a variety of cells, such as hepatoma cells, erythroleukemic cells, and fibroblast-like synoviocytes [11, 12]. Similar effects can be found in our study, in which LMT-28 reduced the expression level of IL-6 in a T2DM rat model with peri-implantitis. Studies have shown that other inflammatory factors associated with peri-implantitis are also affected by LMT-28. Park et al. reported that LMT-28 markedly decreased serum levels of tumor necrosis factor-*α* (TNF-*α*) and interleukin-1*β* (IL-1*β*) in mice with arthritis [12, 26]. In addition, Hong et al. reported that the TNF-*α* titer in mouse serum induced by IL-6 was reduced significantly by LMT-28 [11]. In the progression of pancreatitis in mice, LMT-28 treatment also reduced the expression of the proinflammatory cytokines IL-1*β* and TNF-*α* [11]. Furthermore, the levels of IL-1*β* and TNF-*α* in lung tissues from mice with sepsis were markedly decreased by LMT-28 [28]. As inflammatory biomarkers, the variations of IL-1*β*, IL-6, and TNF-*α* are the most commonly studied functional polymorphisms for peri-implantitis and peri-implant bone loss [18, 33, 34]. Thus, the multiple effects of LMT-28 are anticipated to be appropriate for tissue repair in many bone-related diseases and organs.

In the present study, IL-6-mediated inhibition of RANKL-induced osteoclast formation was achieved by LMT-28, which interfered with IL-6/IL-6R/GP130 trans-signaling. Moreover, the combination of IL-6/IL-6R and GP130 in osteoblasts, which subsequently activated the JAK signaling pathway and preferentially induced tyrosine phosphorylation of STAT3, also correlated with RANKL levels. The JAK2/STAT3 signal transduction pathway is one of the main pathways through which IL-6 exerts its biological roles and plays a critical part in inflammatory bone disease [33]. To study the role of the JAK2/STAT3 signaling pathway in the expression of RANKL, we used LPS, AGEs, LMT-28, and the JAK2 inhibitor baricitinib to treat osteoblasts, and we observed that LPS and AGEs stimulated the expression of p-STAT3, p-JAK2, and RANKL, which were inhibited by baricitinib and LMT-28. Recent studies have found that JAK inhibitors impeded osteoclast activity and osteoporosis via modulating the RANKL and reactive oxygen species signaling pathways, which indicated the possible role of LMT-28 on JAK-related signaling in osteoclasts [35, 36]. However, the p-GP130 level was not influenced by baricitinib. This implies that the JAK2/STAT3 signaling pathway is a downstream effect of the IL-6/GP130 pathway. Hence, the IL-6/GP130/JAK2/STAT3 signaling pathway may be a therapeutic target, particularly in chronic or aggressive bone metabolic diseases.

One limitation of the current study is that, although the SD rat model is an ideal animal model for T2DM and peri-implantitis, it does not completely recapitulate the characteristics of T2DM and peri-implantitis in humans and shows different immune responses. Furthermore, LMT-28 inhibits the secretion of RANKL in osteoblasts through the IL-6/GP130 signaling pathway, but the effect of LMT-28 on osteoclast differentiation and maturation remains to be demonstrated. Studies reported that inflammatory bone resorption was related to RANKL- and LPS-mediated NF-*κ*B in osteoclast, which may indicate the potential mechanism of LMT-28 on bone resorption [37]. RANKL is derived from a variety of cell types in bone tissue, including osteoblasts and osteocytes. The influence of LMT-28 on RANKL, secreted by osteocytes, remains unknown.

## 5. Conclusions

Our study highlighted the potential of LMT-28 to reduce LPS-induced bone resorption around implants in a diabetic rat model with peri-implantitis. LMT-28 also significantly inhibited the mRNA and protein expression of RANKL in osteoblasts under costimulation by LPS and AGEs. These findings provide a rationale for the critical role of IL-6 in LPS-induced bone resorption and in vitro and in vivo evidence of LMT-28 therapeutic effects on peri-implantitis in diabetics.

## Figures and Tables

**Table 1:** The blood glucose of SD rats before and after 1% STZ injection (mmol/L).

Time (d)	Mean	SD	Min	Max	Test value
0	8.52	0.79	7.1	9.8	*F* = 228.77 *p* < 0.001
3	23.71	3.01	19.3	29.2	
7	23.71	3.11	19.1	29.5	
14	23.82	3.32	18.8	29.0	

## Data Availability

The datasets used and/or analyzed during the current study are available from the corresponding author on reasonable request.

## Abbreviations

AGEs: Advanced glycation end products
BAH: Bone absorption height
BAV: Bone absorption volume
TNF-*α*: Tumor necrosis factor-*α*
IL-1*β*: Interleukin-1*β*
IL-60: Interleukin-6
LPS: Lipopolysaccharide
P.g LPS: Porphyromonas gingivalis lipopolysaccharide
T2DM: Diabetes mellitus type 2
PBL: Peri-implant bone loss
RANKL: Receptor activator of nuclear factor-*κ* B ligand
BHI: Brain heart infusion broth
RT-qPCR: Real-time fluorescence quantitative PCR
WB: Western blot
STZ: Streptozotocin
EDTA: Ethylene diamine tetraacetic acid.
